# High Consistency Ramp Design Method for Low Noise Column Level Readout Chain

**DOI:** 10.3390/s24217057

**Published:** 2024-11-01

**Authors:** Zhongjie Guo, Lin Li, Ruiming Xu, Suiyang Liu, Ningmei Yu, Yuan Yang, Longsheng Wu

**Affiliations:** 1 Department of Electronics, Xi’an University of Technology, Xi’an 710048, China; lli@stu.xaut.edu.cn (L.L.); rmxu@stu.xaut.edu.cn (R.X.); syliu@stu.xaut.edu.cn (S.L.); yunm@xaut.edu.cn (N.Y.); yangyuan@xaut.edu.cn (Y.Y.); 2 School of Microelectronics, Xidian University, Xi’an 710071, China; lswu@xidian.edu.cn

**Keywords:** CMOS image sensors, single-slope ADC, adaptive ramp signal generator, correction of inconsistent error in ramp signals

## Abstract

In order to address the inconsistency problem caused by parasitic backend wiring among multiple ramp generators and among multiple columns in large-array CMOS image sensors (CIS), this paper proposes a high-precision compensation technology combining average voltage technology, adaptive negative feedback dynamic adjustment technology, and digital correlation double sampling technology to complete the design of an adaptive ramp signals inconsistency calibration scheme. The method proposed in this article has been successfully applied to a CIS with a pixel array of 8192(H) × 8192(V), based on the 55 nm 1P4M CMOS process, with a pixel size of $10\times 10\;\mu m^{2}$. The chip area is 88(H) × 89(V) ${\mathrm{mm}}^{2}$, and the frame rate is 10 fps. The column-level analog-to-digital converter is a 12-bit single-slope analog-to-digital converter (SS ADC). The experimental results show that the ramp generation circuit proposed in this paper can reduce the inconsistency among the ramp signals to 0.4% LSB, decreases the column fixed pattern noise (CFPN) caused by inconsistent ramps of each column to 0.000037% (0.15 $e^{-}$), and increases the overall chip area and power consumption by only 0.6% and 0.5%, respectively. This method provides an effective solution to the influence of non-ideal factors on the consistency of ramp signals in large area array CIS.

## 1. Introduction

CMOS image sensors (CIS) have been widely used in the automotive industry, security detection, biomedicine, machine vision, and other image processing fields [1]. The image quality of the system is closely related to the resolution of the internal ADC. In order to achieve better performance trade-offs in terms of area, power consumption, accuracy, and conversion speed [2,3,4], most of the internationally popular CIS adopt the processing architecture of single slope ADC (SS ADC) [5,6,7]. The ramp generator is the benchmark of SS ADC quantized data, and the ramp signal with high precision and high linearity is the key to the design of single slope ADC [8,9]. However, due to multiple factors such as layout area, driving ability, and stitching process, the design method of multiple ramps must be adopted for the super large area array CMOS image sensor [10]. At this time, in order to ensure the spatial parameters of the image, it is necessary to ensure the high consistency of ramp generators and column ramp signals.

Generally speaking, the column level ADC provides ramp signals for each column in the way of global ramp [11]. However, with the continuous improvement of the requirements for high-resolution images, the pixel array of image sensors continues to grow [12,13]. In the special application environment of super large area array CIS, there are differences among the ramp signals of each column no matter whether one is using a global ramp signal generator or block-based multi-ramp generators. Of course, the ramp generation circuit itself will also offset the slope of the ramp signal due to the influence of temperature, process, voltage, and other factors, so it is very important to calibrate the ramp circuit adaptively.

The structures of the advanced ramp signal generators reported at present focus on solving the linearity of the ramp and the non-ideal factors inside the ramp. In reference [14], it is proposed to realize the adaptive ramp generator based on negative feedback mechanism through the pulse width lock loop (PWLL) circuit architecture. In the correction process of this scheme, there are comparator delay and digital logic delay, which cause large linearity error. Although the delay of comparator and digital logic in a ramp circuit can be accelerated by the comparator proposed in reference [15], the acceleration is also limited. Even if the problem of delay is solved, the adaptive scheme is to correct the internal error of the ramp generation circuit itself, such as the difference caused by process, power supply voltage, temperature (PVT), so the scheme can not reduce the inconsistency among the ramps of each column under the global ramp or the block-based multi-ramp.

In reference [16], an adaptive ramp circuit based on a negative feedback mechanism is proposed [16,17,18], but the offset voltage of the operational amplifier in the feedback circuit will act on the ramp, resulting in a certain error in the slope. In reference [19], the effect of offset voltage on the ramp is reduced by initializing offset storage of the operational amplifier in the feedback circuit, but even so, the adaptive scheme is also used to correct the internal error of the ramp generation circuit, so it is not suitable for the adaptive correction of inconsistency between the global ramp and the block type multi-ramp.

In reference [20], which focuses on the problem of column level fixed pattern noise caused by the slope difference between the coarse ramp and the fine ramp of the two-step single-slope ADC (TS SS ADC), a scheme of simulating gain error by calibrating the comparator is proposed, which can only be used in the TS SS ADC. In reference [21], in order to reduce the ramp gain error in the ramp input stage, an active ramp input stage scheme is proposed, which converts the absolute error in the ramp gain of the traditional passive ramp input stage into a relative error. However, this scheme still cannot correct the inconsistency problem caused by the parasitic routing in the large array.

In reference [22], the current steering ramp circuit structure is used as the global ramp, and the CFPN of SS ADC is eliminated by digital correlated double sampling (DCDS) [22,23]. However, DCDS technology cannot eliminate the impact of slope offset. In reference [24], a two-step ramp signal generator is implemented by using a resistor-resistor-string digital to analog converter (RRDAC). Through the output offset storage technology, foreground correction, and other technologies, the changes in the offset of the comparator caused by the resistance mismatch of the ramp generator and the input level are eliminated. However, this technology cannot solve the problem that the slope of the ramp signal is distorted by the non-constant MOS capacitor at the input of the comparator in a large array. In reference [25], a noise suppression ramp generator is designed using the current steering ramp circuit structure to improve the noise and linearity of the circuit under low brightness, but this scheme cannot improve the problem that the non-constant MOS capacitor at the input of the comparator distorts the ramp signal in the large array CIS [26]. In reference [27], a two-step ramp circuit is realized through a capacitive DAC (CDAC), and a detachable super CDAC array is connected through the capacitor array of CDAC to improve the linearity. The introduction of super DAC makes the timing of these capacitor array switches more complex, and the accuracy is only 10 bit.

After a large number of literature investigations, the existing ramp generators focus on solving the linearity of the ramp and the non-ideal factors inside the ramp [14,15,16,17,18,19,20,21,22,23,24,25,26,27,28], but even though the linearity of the ramp signal itself can be well guaranteed through the technology in the literature, the parasitic effect of the ramp signal on the transmission line still exists. In the CIS with super large array scale, the use of the above schemes will lead to the problem of column level inconsistency caused by parasitic effects of metal transmission lines [29]. Therefore, this paper studies the ramp inconsistency error and proposes a high consistency ramp design method for low noise column level readout chain.

## 2. Analysis of Influencing Factors of Ramp Inconsistency

**Figure 1:**
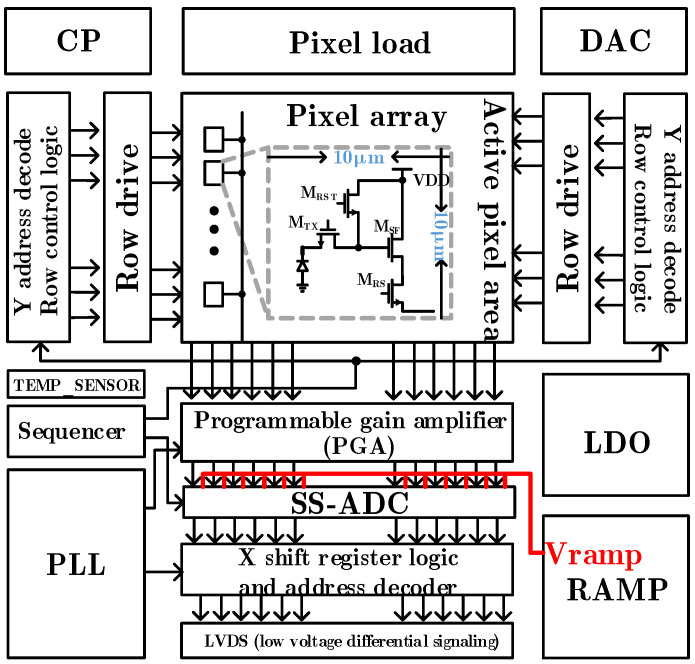
The schematic diagram of CMOS image sensor architecture.

Figure 1 shows the schematic diagram of CMOS image sensor architecture, which includes pixel array, charge pump, row drive module, column level readout circuit, phase locked loop, LVDS (low voltage differential signaling) interface, and other modules. The ramp reference in the column level readout circuit comes from the global ramp voltage signal generated by the ramp generation circuit and provides the ramp signal for each column through the buffer. Currently, the super large-scale CIS have reached the tens of millions or even hundreds of millions of levels, which require nearly tens of thousands of parallel ADCs [30]. Here, if the global ramp signal generator is used to provide the ramp reference for each column, in order to increase the driving capacity of the ramp signal during transmission, the large drive buffer is generally used. However, due to the excessive parasitic draw in the actual routing, the difference of the ramp signals read in each column will be too large. In addition, in the super large area array CIS, tens of thousands of comparators work at the same time, and the non-constant MOS capacitor at the comparator input will distort the ramp signal. These will directly affect the output of each column of ADC digital code and then affect the image quality.

In the case of super large area array CIS, multiple ramp circuits can also be used to provide ramp signals for each column in a partitioned manner. Although it can reduce the influence of line parasitism and the non-constant capacitance of the comparator input stage to a certain extent, there will be natural circuit mismatch among multiple ramp circuits, and the ramp generated by each ramp circuit will be different, resulting in inconsistent ramp reference signals provided to each column.

### 2.1. Analysis of Influencing Factors of Ramp Inconsistency

#### 2.1.1. Influence of Wire Parasitism on Global Ramp Inconsistency

In the super large-scale array CIS, excessive parasitism in the actual routing of the global ramp signal can lead to significant differences in the ramp signals read from each column, directly impacting image quality. The ramp signal varies monotonically and results in varying delays at different column nodes. This can be calculated using the time constant formula, as illustrated in Equation (1):

(1) $$\tau =R_{eq}\cdot C_{eq},$$

where $R_{eq}$ and $C_{eq}$ are the equivalent resistance and equivalent capacitance from the output ramp signal to each node, respectively.

**Figure 2:**
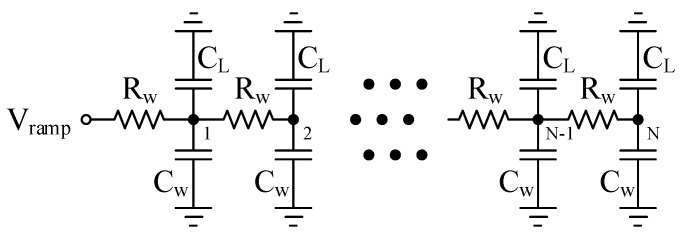
The metal wire distributed RC parasitic model.

The inconsistency of ramp columns is mainly caused by routing parasitism. In order to better restore the principle of routing parasitism, a distributed RC parasitism model is adopted, as shown in Figure 2.

Here, *N* is the number of columns of pixels, *L* is the total length of *N* columns of pixels (that is, the total length of metal wires used to transmit ramp signals), $R_{w}$ is the equivalent resistance of each section of metal wire, $C_{w}$ is the equivalent capacitance of each section of metal wire, and $C_{L}$ is the equivalent load capacitance of each column ramp. Then, according to Equation (1):

(2) $$\tau_{total}=\left(\frac{L}{N}\right)\cdot \left(R_{w}\cdot C+2R_{w}\cdot C+\cdots +NR_{w}\cdot C\right)=\frac{N+1}{2N}\cdot R_{w}\cdot C\cdot L^{2}\approx \frac{1}{2}\cdot R_{w}\cdot C\cdot L^{2},$$

where $C=C_{w}+C_{L}$. In addition, it can be observed from Equation (2) that the total time constant $\tau_{total}$ is approximately proportional to the square of the total length *L* of the metal wire.

**Figure 3:**
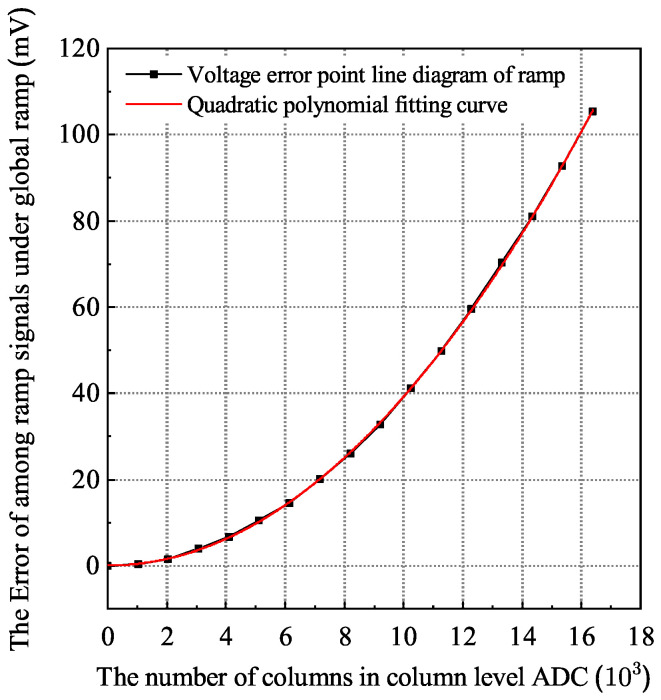
The error curves of different column numbers driven by global ramp circuit.

Based on the 55 nm 1P4M process and the pixel size of $10\times 10\;\mu m^{2}$, the routing length of the global ramp signal under different array sizes is estimated. According to the parasitic parameter analysis results, the circuit simulation model is established. Figure 3 shows the error curve obtained by driving different column numbers by the global ramp circuit. From the simulation results of each column, it can also be obtained that the total error is approximately proportional to the square of the total length *L* of the metal wire, which is consistent with the conclusion obtained by Equation (2).

Through the simulation of the model driving 8192 columns, it is found that the first column and the last column will produce a voltage error of about 26.14 mV under the parasitic environment, which will seriously affect the consistency of the quantization results of each column. Assuming that all parasitic parameters are constant during operation, this error belongs to common mode error among columns, which can be eliminated by digital correlation double sampling technology on columns [31].

#### 2.1.2. Influence of Comparator Input MOS Capacitor on Global Ramp Inconsistency

**Figure 4:**
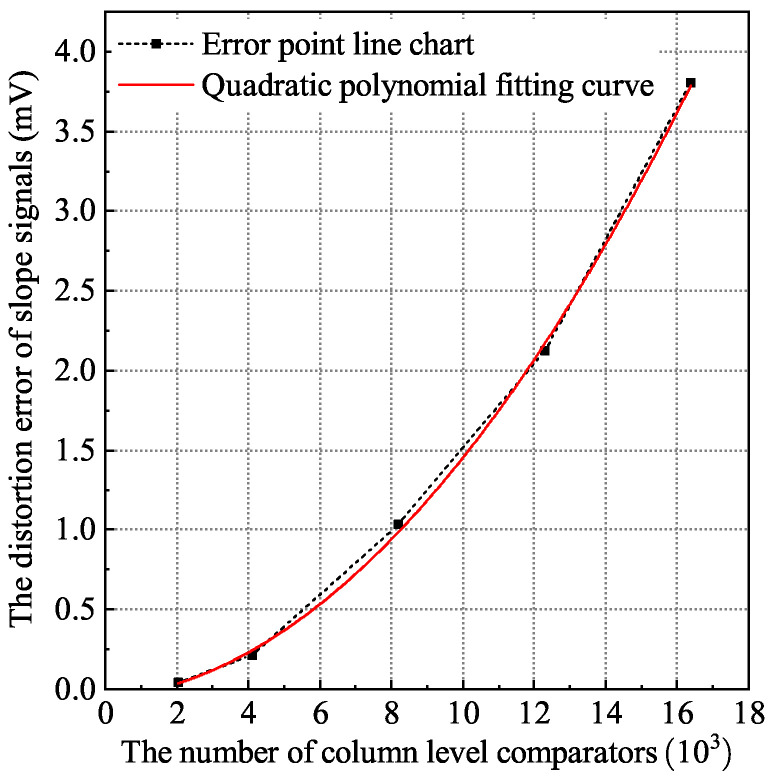
The error curve of the distortion of the non-constant gate capacitance of the comparator input stage to the ramp signal.

In the super large area array CIS, when tens of thousands of comparators work at the same time, there are tens of thousands of non-constant MOS capacitors of comparators that will distort the ramp signal. That is, the $C_{L}$ of each node in the equivalent model in Figure 2 is not constant. From Equation (2), it can be seen that the equivalent $\tau_{total}$ is variable, so the error is a variable error. This variable error cannot be eliminated using the digital correlation double sampling technology, which ultimately makes the linearity of the global ramp signal worse. Figure 4 shows the error curve of the distortion of the ramp signal caused by the non-constant gate capacitance of the comparator input stage. It can be observed that as the array becomes larger and larger, the influence of the non-constant gate capacitance of the comparator input stage on the distortion of the ramp becomes larger and larger. Under the 16 k column array, the error among the columns is most likely to be about 3.8 mV, resulting in an accuracy error of about 10 LSB. In the global ramp mode, these errors will eventually increase the inconsistent noise among the columns.

### 2.2. Analysis on Influencing Factors of Multiple Ramp Inconsistency in Block Type

**Figure 5:**
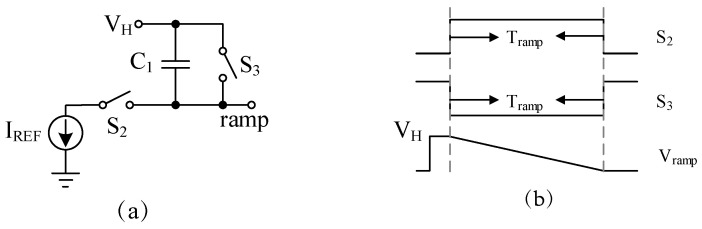
Circuit diagram and working sequence of integral ramp generator: (**a**) circuit diagram; (**b**) sequence diagram.

Assuming that the large area array block type multi-ramp circuits adopt the integrated ramp generator circuit structure as shown in Figure 5, under the working sequence shown in the figure, the output signal of the ramp voltage is as shown in Equation (3). The symbols mentioned in Equation (3) refer to the following meanings: $I_{REF}$ refers to the charging current at the generation stage of the ramp, $T_{ramp}$ refers to the integration time at the generation stage of the ramp, $C_{1}$ refers to the integration capacitance at the generation stage of the ramp, and $V_{H}$ refers to the starting voltage of the ramp. Even if each ramp circuit is guaranteed to be completely consistent in design, the differences in PVT, layout, and other aspects will make the charging current $I_{REF}$, integration time $T_{ramp}$ and integration capacitance $C_{1}$ not exactly equal in each ramp circuit, and can only increase the matching among multi-ramp circuits as much as possible, reduce the differences among them, but cannot be completely eliminated.

(3) $$V_{ramp}=V_{H}-\Delta U=V_{H}-\frac{Q}{C}=V_{H}-\frac{I_{REF}\cdot T_{ramp}}{C_{1}},$$

Therefore, if we want to reduce the influence of parasitic routing and the non-constant capacitance of the comparator input stage by using the multi-ramp method under the large area array CIS, we must solve the problem of matching among the multi-ramp circuit, including the matching of the circuit itself and the matching of the global signal.

The distributed multi-ramp circuit used in this paper interconnects the ramps through metal wires, which can average the ramp voltage signals, reduce the inconsistency caused by the matching problem of the circuit itself, and greatly improve the consistency among the ramp signals. It is very difficult for some global signals to be completely consistent under a large array, especially the ramp start voltage $V_{H}$ here. The global signal mismatch factor introduced here can be eliminated using the partitioned driving method combined with the digital correlation double sampling technology. In addition, in order to realize the adaptive adjustment of distributed multiple ramps, this paper proposes a high consistency ramp design method for low-noise column level readout chains applied to large array CMOS image sensors.

## 3. Adaptive Switched Capacitor Ramp Generator Based on Average Voltage and Voltage Controlled Oscillator

### 3.1. Design of Average Voltage for Distributed Multi-Ramp Circuits

**Figure 6:**
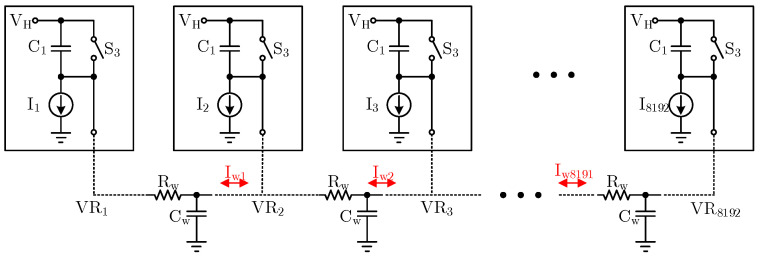
The voltage averaging principle diagram of distributed multiple ramp signal generator.

In this paper, in the array scale of 8192 × 8192 in aerospace applications, a super large array infrared detector with a pixel array of 8192 × 8192 can recognize finer temperature differences and target details, providing clearer and more delicate infrared images. A total of 8192 columns of ramp circuits are required to adopt the distributed ramp design. The 8192 columns are divided into four large modules (the specific blocking method is divided into four blocks according to “from column 0 to column 2047, from column 2048 to column 4095, from column 4096 to column 6143, and from column 6144 to column 8191”), and each large module has 2048 ramp circuits. The 2048 ramp generation circuits are interconnected by metal wires, as shown in Figure 6. In the figure, $R_{w}$ and $C_{w}$ refer to the parasitic resistance and parasitic capacitance of the metal wires among the ramps of each column, respectively.

In the actual circuit, because of the mismatch among current mirrors, the integral current of each column is not uniform, which leads to the different ramp voltages generated on each column at the same time. If the output terminals of each ramp circuit are connected through metal wires, there will be current flow between adjacent ramp circuits due to different ramp voltages at the same time. Under the regulation of the current, the ramp voltages between adjacent ramp circuits are very close. For example, if $I_{1}$ is slightly larger than $I_{2}$, the ramp signal $VR_{1}$ generated at the same time is slightly smaller than $VR_{2}$ at the ramp generation stage. At this time, there will be a current flowing from $VR_{2}$ to $VR_{1}$. Under the action of this current, $VR_{1}$ and $VR_{2}$ will eventually be average and nearly equal. The same is true for the average principle on other columns. Taking 2048 ramps as a large unit, the ramp signals generated by the 8192 ramp circuits are finally averaged regionally, so as to reduce the difference among the columns. In addition, the average voltage scheme can transform the matching among the current mirror devices in each column into the overall matching among the large units, which makes it easier to achieve a high degree of consistency of the integral signals in each column.

**Figure 7:**
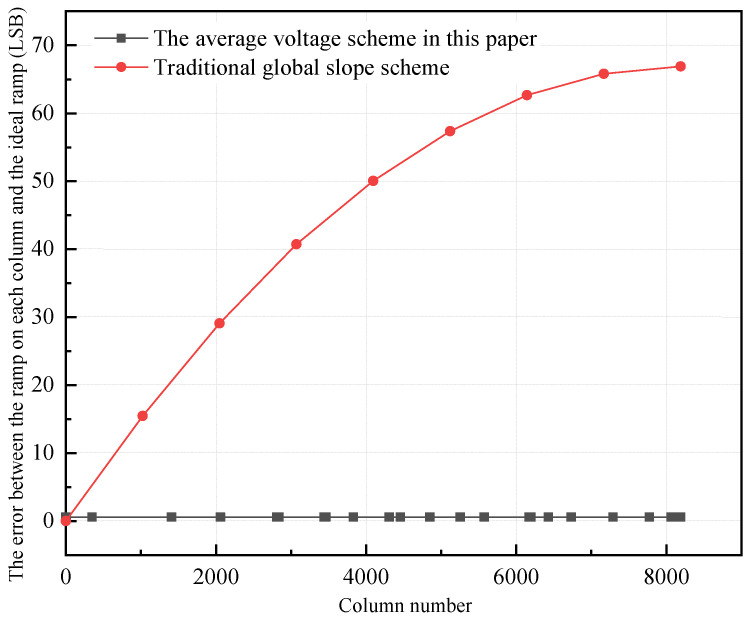
Column level inconsistency error caused by ramp inconsistency between the average voltage scheme and the traditional global ramp scheme.

As shown in Figure 7, the traditional global ramp scheme will bring a maximum ramp error of about 67 LSB at the 8192nd column ramp; after using the average voltage scheme in this paper, the ramp error of each column is almost 0.59 LSB, which can better highlight the ramp consistency advantage of the average voltage scheme.

### 3.2. High Consistency Adaptive Ramp Circuit for Large Area Array CMOS Image Sensors

**Figure 8:**
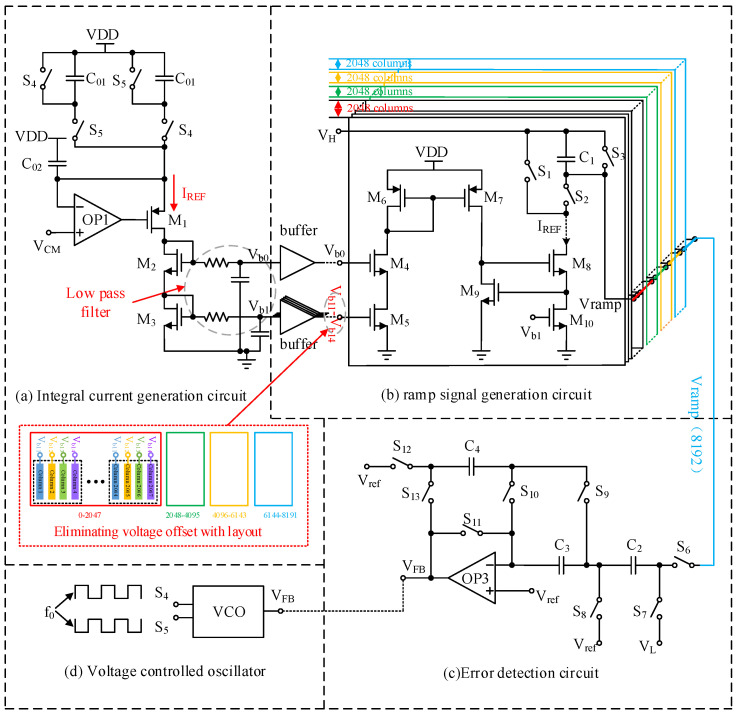
The high consistency adaptive ramp circuit for a large area CMOS image sensor.

As shown in Figure 8, the high consistency adaptive ramp circuit of a large area CMOS image sensor is composed of four main modules: integral current generation circuit, ramp signal generation circuit, error detection circuit, and voltage controlled oscillator.

The integrated current generation circuit copies the generated integrated current to the 8192 columns distributed ramp signal generation circuit through the cascode current mirrors, and the bias voltages $V_{b0}$ and $V_{b1}$ increase the driving capacity through the buffers. Among them, $V_{b1}$ is converted into $V_{b11}$, $V_{b12}$, $V_{b13}$, and $V_{b14}$ after passing through four buffers, and the voltage offset is eliminated using the layout. The integrated current generation circuit is a voltage to current conversion realized using switched capacitors to simulate accurate resistors, as shown in Figure 8a. The principle of its current generation is the same as Equation (4).

(4) $$I_{REF}=\frac{V_{DD}-V_{CM}}{\frac{1}{f_{0}\cdot C_{01}}}=\left(V_{DD}-V_{CM}\right)\cdot f_{0}\cdot C_{01},$$

The value of $I_{REF}$ is determined by the control signal frequency $f_{0}$ of switches $S_{4}$ and $S_{5}$, common mode voltage $V_{CM}$, and capacitance $C_{01}$. Due to the switching of the switching capacitor, the voltage fluctuation of $M_{1}$ drain stage will still be coupled to the gate of the cascode current mirror through the parasitic capacitance of the MOS device, which is not conducive to the stability of the current and the improvement of the noise performance of the ramp generated circuit. In order to ensure the stability of the generated current, a low-pass filter is added to the grid of the cascode current mirror.

Figure 8b is a ramp signal generation circuit. In the case of large array CIS, the driving capacity of the ramp start voltage $V_{H}$ is very high. Therefore, four buffers are used to provide start voltage $V_{H}$ for 8192 columns. Considering that the offset voltage among each buffer is different, common mode error will be introduced into the ramp circuit. Therefore, the common mode differences need to be eliminated by combining digital correlation double sampling technology in the design. In Figure 8b, $M_{8}$, $M_{9}$, and $M_{10}$ constitute a modulated cascode current mirror. Compared with the common cascode current mirror, this structure has higher output impedance, so it is more conducive to improve the linearity of the ramp signal. The error detection circuit in Figure 8c only samples the ramp output signal of the last column of ramp voltage generation circuits and feeds back the difference between the termination voltage of the actual ramp and the termination voltage $V_{L}$ of the ideal ramp to the $V_{FB}$ signal. In Figure 8d, $V_{FB}$ is the input terminal of the voltage controlled oscillator. After feedback, the $V_{FB}$ controls the voltage controlled oscillator to generate a digital signal with a frequency of $f_{0}$, which is the switch control signal in the integral current generation circuit. The frequency $f_{0}$ completes the negative feedback mechanism of adaptive adjustment of ramp by adjusting the required integral current.

**Figure 9:**
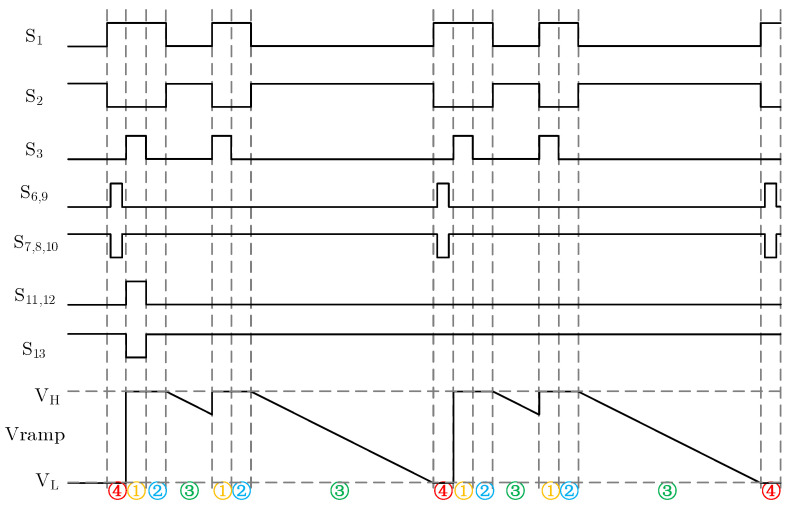
The sequence diagram of adaptive ramp circuit.

As shown in Figure 9, the sequence diagram of the adaptive ramp circuit can be summarized into four processes: ➀ reset, ➁ hold, ➂ ramp generation, and ➃ calibration. In addition, $V_{FB(1)}$–$V_{FB(4)}$ refer to the voltage signal $V_{FB}$ of the circuit in the reset stage, hold stage, ramp generation stage, and calibration stage, respectively. Because there are differences between each stage, they can be distinguished. The specific work process is as follows:

➀ Reset stage: switches $S_{1}$, $S_{3}$, $S_{7}$, $S_{8}$, $S_{10}$, $S_{11}$, and $S_{12}$ are on, while $S_{2}$, $S_{6}$, $S_{9}$, and $S_{13}$ are off. The charge on capacitor $C_{1}$ can be expressed as

(5) $$Q_{C1(1)}=\left(V_{H}-V_{H}\right)\cdot C_{1},$$

The charge on capacitor $C_{2}$ can be expressed as

(6) $$Q_{C2(1)}=\left(V_{L}-V_{ref}\right)\cdot C_{2},$$

Suppose that the operational amplifier in the error detection circuit has the offset voltage of $V_{OS}$, the capacitor $C_{3}$ is the offset elimination capacitor, and the charge of $C_{3}$ is

(7) $$Q_{C3(1)}=\left[V_{ref}-\left(V_{ref}-V_{OS}\right)\right]\cdot C_{3}=V_{OS}\cdot C_{3},$$

The charge on capacitor $C_{4}$ is

(8) $$Q_{C4(1)}=\left[V_{FB(1)}-V_{ref}\right]\cdot C_{4},$$

In addition, the calibration circuit only resets once in the whole working process; that is, it only stores the offset in the first reset stage.

➁ Hold stage: switches $S_{1}$, $S_{7}$, $S_{8}$, $S_{10}$, and $S_{13}$ are on; $S_{2}$, $S_{3}$, $S_{6}$, $S_{9}$, $S_{11}$, and $S_{12}$ are off. The charge on capacitor $C_{1}$ can be expressed as

(9) $$Q_{C1(2)}=\left[V_{ref}-V_{ramp(2)}\right]\cdot C_{1},$$

The charge on capacitors $C_{2}$ and $C_{3}$ in the hold stage is consistent with that in the reset phase. At this time, $V_{FB(2)}=V_{FB(1)}$, the charge on capacitor $C_{4}$ is

(10) $$Q_{C4(2)}=\left[\left(V_{ref}-V_{OS}\right)-V_{FB(2)}\right]\cdot C_{4},$$

➂ Ramp generation stage: switches $S_{2}$, $S_{7}$, $S_{8}$, $S_{10}$, and $S_{13}$ are on; $S_{1}$, $S_{3}$, $S_{6}$, $S_{9}$, $S_{11}$, and $S_{12}$ are off. The switching state of the error detection circuit in this stage is exactly the same as that in the hold stage, and the charges of capacitors $C_{2}$, $C_{3}$, and $C_{4}$ are the same as that in the hold stage. The formula for generating ramp voltage signal at this stage is

(11) $$V_{ramp}=V_{H}-\frac{I_{REF}\cdot T_{ramp}}{C_{1}}=V_{H}-\frac{\left(V_{DD}-V_{CM}\right)\cdot f_{0}\cdot C_{01}\cdot T_{ramp}}{C_{1}},$$

where $T_{ramp}$ is the integration time corresponding to the ramp generation signal, and $C_{1}$ is the integration capacitance. It can be seen from Equation (11) that, within the integration time of $T_{ramp}$, the circuit will generate a descent ramp with an initial level of $V_{H}$ as time goes by. Expressions $C_{01}$ and $C_{1}$ in Equation (11) are relative quantities, and this ratio relationship can be well maintained in the actual process; $f_{0}$ is the frequency of the switch control signal of the integral current generation module; $f_{0}$ and $T_{ramp}$ are both digital signals, so the impact is also small.

➃ Calibration stage: integration stops, switches $S_{1}$, $S_{6}$, $S_{9}$, and $S_{13}$ are on, and $S_{2}$, $S_{3}$, $S_{7}$, $S_{8}$, $S_{10}$, $S_{11}$, and $S_{12}$ are off. During the ramp generation stage, the termination voltage $V_{ramp(3)}$ of the actual ramp is sampled on the capacitor $C_{2}$, and the charge on the capacitor $C_{2}$ can be expressed as

(12) $$Q_{C2(4)}=\left[V_{ramp(3)}-V_{ref}\right]\cdot C_{2},$$

The charge of capacitor $C_{3}$ is also the charge saved in the reset stage:

(13) $$Q_{C3(4)}=V_{OS}\cdot C_{3},$$

The charge on capacitor $C_{4}$ is

(14) $$Q_{C4(4)}=\left[V_{ref}-V_{FB(4)}\right]\cdot C_{4},$$

From the ramp generation stage to the calibration stage, the right pole plate of capacitor $C_{2}$ and the left pole plate of $C_{4}$ meet the charge conservation, then:

(15) $$-Q_{C2(3)}+Q_{C4(3)}=-Q_{C2(4)}+Q_{C4(4)},$$

$$-\left(V_{L}-V_{ref}\right)\cdot C_{2}+\left[\left(V_{ref}-V_{OS}\right)-V_{FB(2)}\right]\cdot C_{4}=-\left[V_{ramp(3)}-V_{ref}\right]\cdot C_{2}+\left[V_{ref}-V_{FB(4)}\right]\cdot C_{4},$$

After simplification, Equation (16) can be obtained:

(16) $$V_{FB(4)}=\frac{C_{2}}{C_{4}}\cdot \left[V_{L}-V_{ramp(3)}\right]+V_{FB(2)}+V_{OS},$$

As can be seen from Equation (16), the adaptive calibration circuit samples the actual termination output voltage of the ramp after the end of each ramp generation cycle, compares it with the termination voltage $V_{L}$ of the ideal ramp, and sends the difference of the comparison to the input $V_{FB}$ of the voltage controlled oscillator through the error detection circuit, so that the control signal frequency $f_{0}$ of switches $S_{4}$ and $S_{5}$ can be adjusted accordingly, so as to change the size of the integral current and realize the slope adaptive calibration of the ramp signal.

## 4. Results and Analysis

In order to verify the feasibility of the method proposed in this paper, the method is applied to a CMOS image sensor with 8192(H)×8192(V) pixel array based on 55 nm 1P4M process, and the performance and inconsistency error correction effect of the distributed integral ramp generation circuit proposed in this paper are simulated and verified. The following two methods are mainly used to verify the effect of ramp inconsistency error correction: ➀ the scheme to test the correction effect of ramp circuit directly starts from the ramp circuit, directly checks the output waveform of each ramp circuit, and obtains whether the slope of each ramp meets the error requirements. ➁ The scheme for checking the correction effect of ramp circuit can also quantize the same analog value through the SS ADC on each column to check the digital code error.

**Figure 10:**
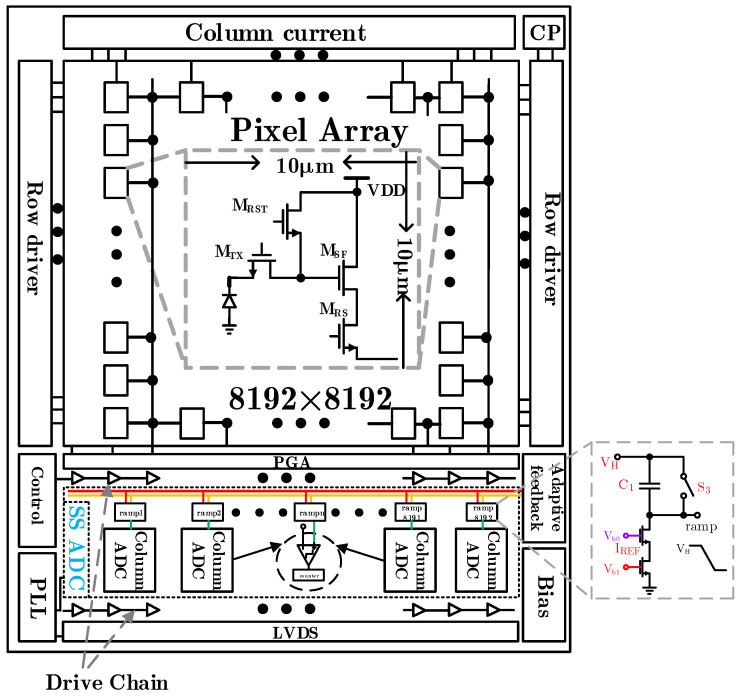
The schematic diagram of the overall layout.

Figure 10 shows the layout of a complete CMOS image sensor chip with a pixel array of size 8192(H)×8192(V). In order to ensure sufficient driving ability of each signal, all global signals are completed through driving ability enhancement technology. The ramp signal here is generated by the voltage averaging method of the distributed multi ramp signal generator as shown in Figure 6, which requires a accurate ramp reset voltage signal $V_{H}$ for the 8192 column ramp generators.

**Figure 11:**
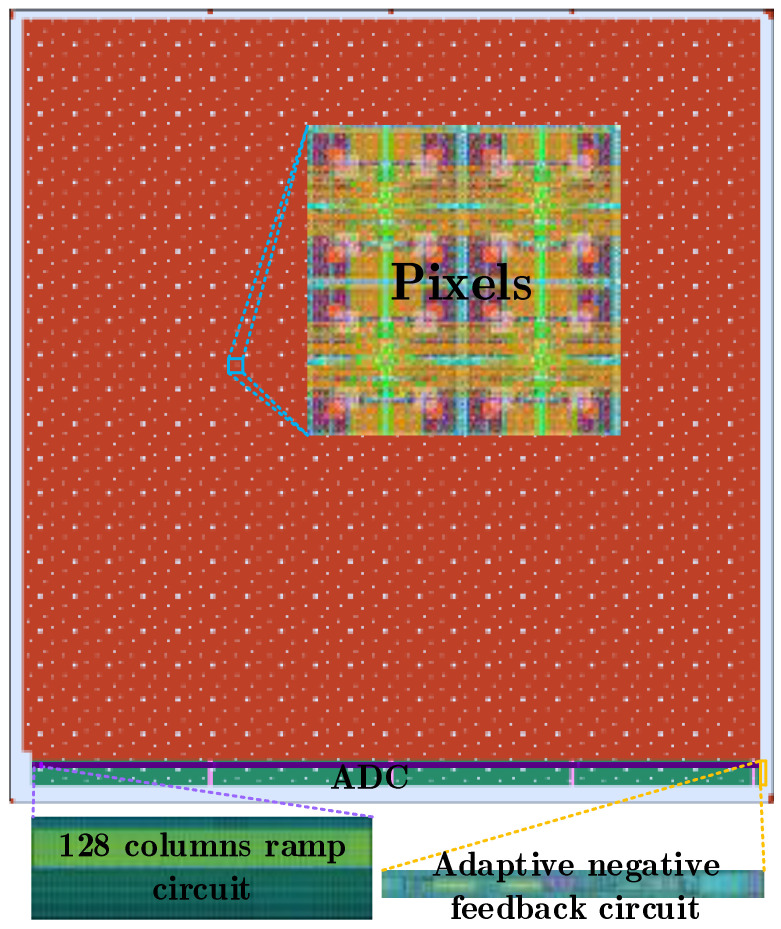
Overall layout design.

In order to reduce the influence of the difference of ramp reset voltage $V_{H}$ caused by excessive parasitism under the large array, four buffers are used to drive 8192 columns in a block mode. Considering that the difference among the four buffers will lead to different common mode differences among the modules, it is necessary to eliminate the common mode difference through digital correlation double sampling technology. At the bottom left of the layout is the adaptive negative feedback circuit, which provides the bias voltage of the integral current for each column of ramp signal generator. The overall layout of the CMOS image sensor is shown in Figure 11.

**Figure 12:**
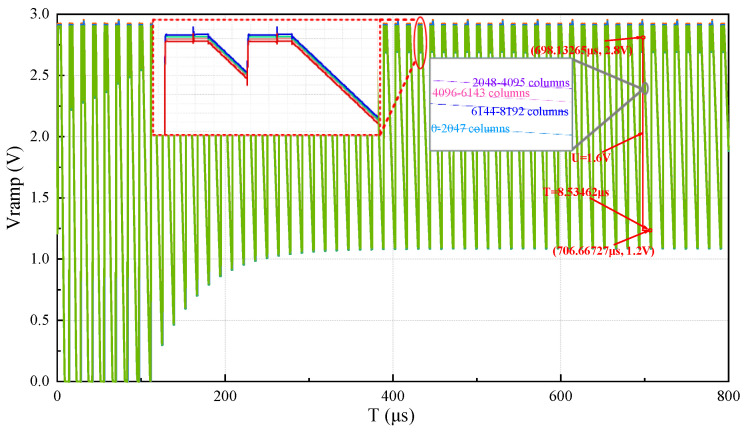
The adaptive calibration waveform of the ramp signal generator.

It can be seen from Figure 12 that, at first, the ramp signals are constantly adjusted in the direction of the ideal ramp, and finally the ramps reach stability after 0.7 ms. The stability time of the ramp signals is related to the ratio of the sampling capacitor $C_{2}$ and the feedback capacitor $C_{4}$ and the ramp signal period. If the ramp signal period decreases, the corresponding time to reach the stable state will decrease. Figure 12 illustrates the ramp voltage adjusted by the adaptive ramp generator. When the output swing is 2.8 V–1.2 V and U = 1.6 V, T = 8.53462 μs (when U is 1.6 V, the ideal ramp is T = 8.53333 μs), that is, the ramp generator has realized the function of slope adaptive adjustment. The ramp generation circuit will choose a period of time after the system power on initialization is completed in actual use, which is enough to make the ramp signals reach a stable state, so the speed of SS ADC will not be affected by the existence of ramp adaptive calibration process in actual work.

**Figure 13:**
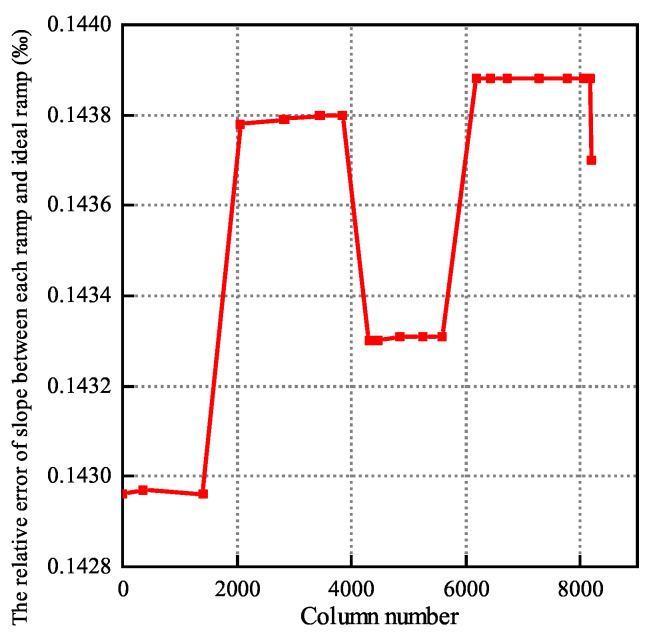
The relative error diagram of slope between adaptive ramp and ideal ramp.

As shown in Figure 13, it can be seen that the difference between the maximum value and the minimum value of the actual ramps and the ideal slope is about 0.144% and 0.143%, respectively. By making a difference between the maximum value and the minimum value, it can be concluded that the slope difference among the ramp signals of 8192 columns is only one millionth, which obviously ensures high consistency of the ramp signal. There is less than 0.144% error between the slope and the ideal slope of 0.1875 V/μs.

**Figure 14:**
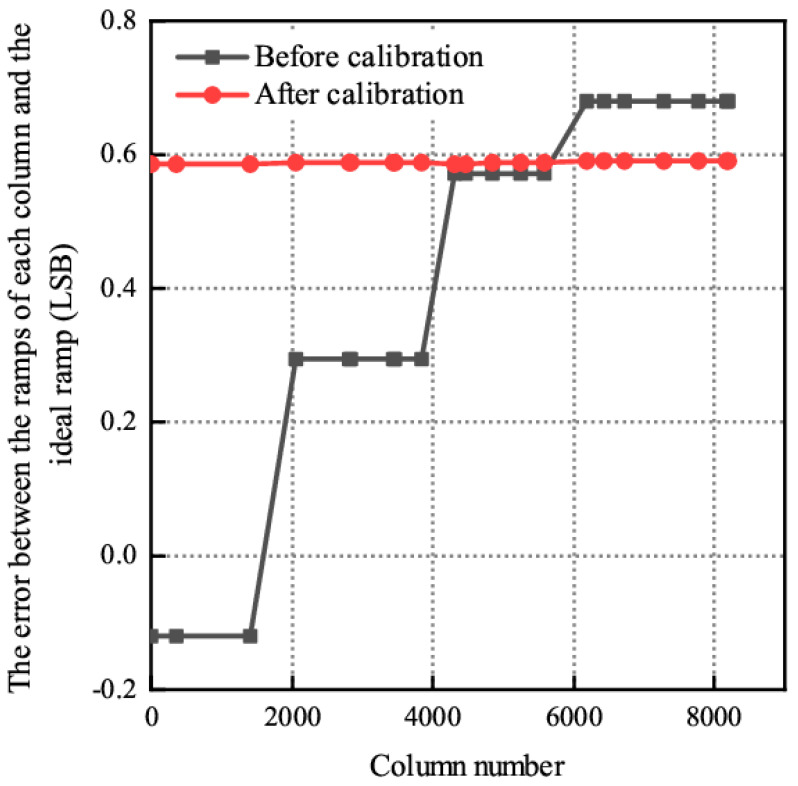
The error diagram among the ramp signals of each column.

In Figure 8a in the integrated current generation circuit, because the switched capacitor is used to simulate the precise resistance, the integrated current has a small fluctuation Δi due to the switching frequency $f_{0}$ when generating the current. Here, if the low-voltage cascode circuit is used to generate the bias voltage $V_{b1}$, noise of $\Delta i\cdot g_{m}\cdot r_{o}^{2}$ will be introduced into the $V_{b1}$. The existence of $\Delta i\cdot g_{m}\cdot r_{o}^{2}$ will make $V_{b1}$ have a large fluctuation. The transmission of the fluctuating $V_{b1}$ from the 8192nd column to the first column will have a delay in time, resulting in a regular difference between the ramp integrated current and the distance of each column, resulting in the inconsistency of ramp signals among each large module. As shown in Figure 14, the point line diagram before correction is the curve diagram corresponding to the bias voltage provided by the low-voltage cascode circuit, and the maximum error among each large module is about 0.8 LSB. In order to reduce the introduction of noise here, the ordinary cascode circuit is used to generate $V_{b1}$. At this time, the noise introduced above the voltage of $V_{b1}$ is only $\Delta i\cdot r_{o}$. Combined with the modulated cascode current mirror, the integral current is copied to each column of ramp circuit to further improve the linearity of the ramp signal, as shown in the corrected dotted line diagram in Figure 13. After using the above method, the inconsistency of ramp signals among each large module is reduced by 99.2%, and the maximum error generated is less than 0.4% LSB, which greatly improves the consistency of each ramp.

**Figure 15:**
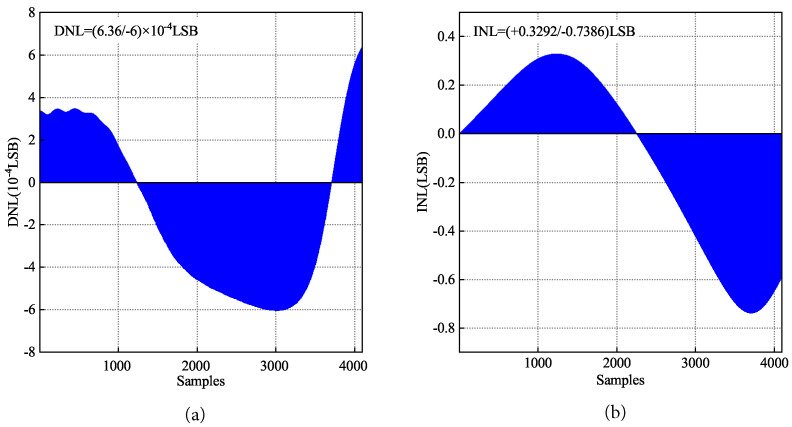
Nonlinear error diagram of ramp signal of 12 bit adaptive ramp generator: (**a**) DNL; (**b**) INL.

Discrete sampling of 4096 ($2^{12}$) points is performed on the generated ramp signal, and the DNL and INL of 4096 data points obtained from sampling are calculated and analyzed using MATLAB software. The results are shown in Figure 15. In order to prevent code loss, the ideal design requirement of DNL is that its value is in the range of ±0.5 LSB. From Figure 15a, DNL is +0.000636 LSB/−0.0006 LSB, which meets the design requirements. From Figure 15b, INL is +0.3292 LSB/−0.7386 LSB.

Table 1 compares the performance parameters of these design methods with those of references [10,12,29,30]. Although there are differences in pixel size and pixel resolution in the literature, the size of wire parasitism can be measured by the size of chip area, and the number of column level comparators can be determined by the number of columns of pixels. Compared with references [10,12,29,30], the pixel unit in this paper has a large full well capacity. The CMOS image sensor in this project is an infrared detector for aerospace. The size of a single pixel unit is 10 × 10 $\mu m^{2}$, which is much larger than the pixel unit in other studies, the integral capacitance in the pixel can be large, and a large pixel integral capacitance can obtain a larger full well capacity. Compared with references [10,30], the CFPN caused by the inconsistency among the ramps of each column is reduced by more than 99.8%. In conclusion, the design method in this paper can explain that, in the application of a super large area array CMOS image sensor, the CFPN caused by the inconsistency among the ramps of each column can be greatly reduced only by increasing the area by 0.6% and the power consumption by 0.5%. When using the traditional method, the chip area is 88 mm(H) × 88.5 mm(V), and the chip length in the vertical direction is 88.5 mm. However, when the chip uses the method in this paper, it introduces an additional length of about 500 μm in the vertical direction, so it will introduce 0.6% more in the overall area. In the traditional method, each column of the circuit needs to consume about 75 μA of current. After using the method in this paper, it needs to consume about 360 nA more current in each column, so the power consumption on each column is about 0.5% more.

## 5. Conclusions

At present, the scale of the high-end image sensor array has reached tens of millions or even hundreds of millions. With the increasing scale of the image sensor array, the parasitic metal routing on the ramp becomes larger and larger, and the inconsistency of the ramp becomes more and more obvious. Tens of thousands of comparators work at the same time, and the distortion of the ramp signal of the input stage of the comparator is becoming more and more serious, resulting in the failure of the global ramp. Although the use of regional multi-ramp design can reduce the above effects, there is a natural mismatch among multi-ramp circuits, which aggravates the inconsistency of ramp signals. In this paper, a design method of a high consistency adaptive ramp circuit based on distributed integration is proposed. The method is applied to a CMOS image sensor with 8192(H) × 8192(V) pixel array using 55 nm 1P4M process. The pixel size is 10 × 10 $\mu m^{2}$, the chip area is 88(H) × 89(V) ${\mathrm{mm}}^{2}$, and the analog-to-digital converter is a 12bit-SS ADC. The experimental results show that the column fixed mode noise caused by the inconsistent ramps of each column can be reduced to 0.000037% at a frame rate of 10 fps, the inconsistency error among the ramp signals of each column is less than 0.4% LSB, and the chip area and power consumption are only increased by 0.6% and 0.5%, respectively. This method provides a theoretical guidance for the development of high-performance large area array CMOS image sensors.

## Figures and Tables

**Table 1:** Performance comparison among different ramp generation circuits.

Performance	[10]	[12]	[29]	[30]	This Work
Process	65 nm 1P4M	90 nm CMOS	130 nm 1P4M	65 nm CMOS	55 nm 1P4M
Pixel resolution	26,456 (H) × 15,072(V)	35.6 Mpix	19,712(H) × 12,752(V)	12,000(H) × 9000(V)	8192(H) × 8192(V)
Pixel size $\left(\mu m^{2}\right)$	3.9 × 3.9	1.22 × 1.22	1.5 × 1.5	0.7 × 0.7	10 × 10
Chip area $\left({\mathrm{mm}}^{2}\right)$	101.84(H) × 58.50(V)	11.4(H) × 10.5(V)	32.84(H) × 25.84(V)	—	88(H) × 89(V)
ADC	14bits-SS ADC	10-bits-SS ADC	12bits-SS ADC	10bits-SS ADC	12bits-SS ADC
Frame rate	1 fps	59 fps	5 fps	10 fps	10 fps (50 fps@HMD)
Temporal noise	3.7 $e_{rms}^{-}$	1.57 $e_{rms}^{-}$	7.1 $e_{rms}^{-}$	1.4 $e_{rms}^{-}$s	5.4 $e_{rms}^{-}$
Full well capacity	31.5 k $e^{-}$	7.773 k $e^{-}$	7.55 k $e^{-}$	—	400 k $e^{-}$
CFPN	0.028% (8.8 $e^{-}$)	—	—	0.00467%	0.000037% (0.15 $e^{-}$)

## Data Availability

The data presented in this study are available on request from the corresponding author. The data are not publicly available due to privacy.
